# The miR-143/145 cluster in prostate cancer: molecular regulation, context-dependent functions, and medical implications

**DOI:** 10.1186/s13062-026-00776-6

**Published:** 2026-04-11

**Authors:** Matthias B. Stope, Holger H. H. Erb

**Affiliations:** 1 Division for Physical Plasma Medicine, Department of Gynecology and Gynecological Oncology, University Hospital Bonn, Venusberg-Campus 1, 53127 Bonn, Germany; 2 Department of Urology, Faculty of Medicine, University Hospital Carl Gustav Carus, Technische Universität Dresden, Fetscherstraße 74, 01307 Dresden, Germany

**Keywords:** RAS MAPK signaling, Epithelial-mesenchymal transition, Tumor microenvironment, KRAS regulation, MicroRNA replacement therapy, Biomarker signature, Biochemical recurrence

## Abstract

The microRNA (miR) cluster miR-143/145 represents a well-characterized tumor-suppressive regulatory system with a multifaceted role in prostate cancer. Both miRs are consistently downregulated during disease progression, and their loss is associated with enhanced proliferation, invasion, epithelial–mesenchymal transition, and metastatic competence. Mechanistically, the cluster modulates Rat Sarcoma Viral Oncogene Homolog (RAS)-Mitogen Activated Protein Kinase (MAPK) signaling via Kirsten Rat Sarcoma Viral Oncogene Homolog (KRAS) and Extracellular Signal-Regulated Kinase 5 (ERK5), Tumor Protein p53 (p53)-dependent growth control through MYC Proto-Oncogene, Basic Helix-Loop-Helix Transcription Factor (c-MYC) repression, apoptosis via B-Cell Lymphoma 2 Interacting Protein 3 (BNIP3), and cytoskeleton-associated motility factors including Fascin Actin-Bundling Protein 1 (FSCN1), Human Enhancer of Filamentation 1/ Neural Precursor Cell Expressed, Developmentally Down-Regulated Protein 9 (HEF1/NEDD9), Golgi Membrane Protein 1 (GOLM1), and Fibronectin Type III Domain Containing 3B (FNDC3B). Downregulation is mainly driven by p53 dysfunction, promoter methylation, and RAS-dependent transcriptional repression. A defining feature is pronounced cell-type specificity, with tumor-suppressive effects in epithelial cells and context-dependent pro-angiogenic functions in stromal compartments, with direct translational relevance. Clinically, miR-143/145 contribute to multimarker diagnostic signatures, while reduced miR-145 correlates with adverse pathology and biochemical recurrence. Preclinical replacement strategies reduce tumor growth and enhance docetaxel sensitivity, yet context-dependent effects necessitate cell type-specific delivery. Overall, the cluster represents a central regulator with diagnostic, prognostic, and therapeutic potential requiring prospective validation.

## Introduction

The miRs miR-143 and miR-145 form a functionally coupled cluster with tumor-suppressive activity in numerous solid malignancies, including prostate cancer. Early investigations demonstrated consistent downregulation of both miRs in epithelial cancers, associated with increased proliferation, enhanced invasiveness, and reduced apoptosis [1]. These antitumor effects were confirmed in independent functional models [2]. The cluster further suppresses central metabolic and growth signaling pathways, reinforcing its tumor-suppressive role [3], and modulates cellular stress responses, thereby limiting malignant phenotypes [4].

In uro-oncology, miR-145 and, to a lesser extent, miR-143 have been identified as tumor suppressors and diagnostic markers. Both miRs discriminate prostate carcinoma from benign tissue [5] and contribute to diagnostic and prognostic signatures [6]. Downregulation was confirmed in independent cohorts, and a systematic review highlights the particular clinical relevance of miR-145. In a multiplatform miR profiling study of prostate carcinoma, Wach et al. analyzed tumor and benign prostate tissues from an independent patient cohort and confirmed significantly reduced expression of both miR-143 and miR-145 using complementary profiling approaches, including microarray-based screening and quantitative validation methods, thereby strengthening the reproducibility of the observed deregulation pattern [7]. Consistent observations have been summarized in a recent review of urologic tumors, which integrates results from multiple study types, including clinical tissue analyses, molecular profiling studies, and experimental functional investigations across different cohorts and platforms, and confirms recurrent downregulation of miR-145 in prostate cancer tissues [8]. Collectively, these analyses support the robustness of miR-143/145 loss as a reproducible molecular feature of prostate cancer.

miRs are evolutionarily conserved, approximately 22-nucleotide non-coding RNAs that regulate gene expression post-transcriptionally. Their biogenesis, involving RNA polymerase II transcription, Drosha/DGCR8 and Dicer processing, and RISC incorporation, has been well characterized [1], with comprehensive overviews available [9]. Target recognition is mediated by sequence complementarity, usually within the 3′-UTR, leading to translational repression or mRNA degradation. The underlying interaction principles and RISC-mediated regulation are detailed in [1, 9, 10].

Genome-wide analyses indicate that a substantial fraction of protein-coding genes is regulated by miRs, which function as fine-tuning modulators within complex networks [1, 10]. Their broad integration into the cellular regulatory architecture and their global roles in development, differentiation, and stress responses have been documented experimentally and systematically [9, 11], consistent with their emergence during multiple evolutionary phases and their linkage to an early RNA-based evolutionary concept [1, 12]. In modern organisms, miRNAs enhance regulatory robustness by reducing noise and stabilizing signaling networks [1, 9, 11].

In tumor biology, miRs exert tumor-suppressive and oncogenic functions. Dysregulation affects proliferation, apoptosis, growth signaling, and adhesion-related networks [3, 4, 13], and core mechanisms of miR-mediated tumorigenesis have been described [12]. In prostate cancer, characteristic diagnostic and prognostic miR signatures were identified [5] and validated independently [14], including relevance for aggressive subtypes [6, 7]. Mechanisms of dysregulation comprise genomic instability [4], promoter methylation [15], and p53-dependent alterations in miR processing and transcription [16, 17]. Functionally, miRs regulate cell cycle and apoptosis [13], IGF1R-associated growth networks [18], epithelial–mesenchymal transition [3], and ERBB3-related adhesion and metastasis pathways [19].

The miR-143/145 cluster exhibits pronounced cell-type specificity, with predominant expression in stromal populations such as smooth muscle cells and fibroblasts [2]. Apparent loss in tumor tissue often reflects stromal alterations, as shown by in situ and laser capture analyses [20], and is discussed in the context of stromal regulation [21]. For diagnostic and therapeutic applications, stromal and epithelial expression must therefore be distinguished [8, 20, 21].

Against this background, the following sections address development, progression, diagnosis, therapy, and prognosis of prostate cancer, focusing on the mechanistic role of the miR-143/145 cluster.

## Biological background of the miR-143/145 cluster

### Genomic localization, transcription, and general functions

miR-143 and miR-145 are organized as a cluster on chromosome 5q32 and are processed from a common primary transcript (pri-miR-143/145). The fundamental genomic organization of this cluster has been described in detail and molecularly defined by multiple independent studies [1, 2, 20].

Both miRNAs regulate a broad spectrum of target genes controlling central cellular processes, including proliferation, differentiation, cell motility, and apoptosis. In diverse experimental tumor models, the miR-143/145 cluster has been characterized as a tumor suppressor that regulates key oncogenic signaling pathways [1, 3, 4, 22]. In the following sections, the specific functional implications of this regulatory system are discussed in the context of prostate cancer and prostate cancer cells.

Functionally, miR-143 targets key components of the RAS-MAPK signaling pathway, including KRAS and ERK5, thereby modulating proliferative and metastatic programs [3, 18, 23, 24].

miR-145 primarily regulates transcription factors such as c-MYC, pro-survival signaling pathways, and cytoskeleton-associated motility proteins, including FSCN1 and HEF1/NEDD9, thereby inhibiting invasive and metastatic phenotypes [17, 22, 25, 26]. Together, both miRs coordinately modulate oncogenic networks that are central to the initiation and progression of prostate cancer.

### Regulation by p53, RAS, and epigenetic mechanisms

Multiple integrated control mechanisms mediate cluster regulation. The transcription factor p53 modulates expression at two levels: it enhances pri-miR processing via p68/Drosha [16] and simultaneously activates transcription of mir-145, thereby contributing to repression of c-MYC [17].

In contrast, the RAS-MAPK signaling pathway directly represses the mir-143/145 promoter through the transcription factor RREB1 [23]. Increased RAS activity amplifies this effect, leading to systematic attenuation of cluster expression [27]. As a consequence, RAS-dependent effectors are derepressed, establishing a tumorigenic feed-forward circuit supported by functional data [4, 23, 27].

Epigenetic mechanisms provide an additional layer of suppression. Promoter methylation at the miR-145 locus, particularly in combination with p53 mutations, results in a marked reduction in miR-145 expression [15].

Thus, the cluster is embedded in a dense regulatory network involving p53-dependent control, RAS signaling, and epigenetic silencing, as substantiated by multiple molecular studies [4, 15, 22, 27].

### Cell-type specificity and tumor microenvironment

Expression of both miRs is highly cell-type specific. In many organs, miR-143/145 are predominantly expressed in stromal cell populations, including fibroblasts, smooth muscle cells, and pericytes, whereas expression in epithelial cells is considerably lower [20, 21]. Notably, the miR-143/145 cluster exhibits a dual role across tumor types. While miR-143 and miR-145 exert tumor-suppressive functions in epithelial tumor cells, increased expression of the cluster in stromal cell populations, such as fibroblasts, has been shown to promote neoangiogenesis and tumor growth in lung cancer [28]. These pro-angiogenic effects contrast with the inhibitory effects observed in epithelial tumor compartments and represent a challenge for miR-based therapeutic strategies in tumors with a prominent stromal component.

In situ hybridization and laser capture microdissection analyses demonstrate that apparent tumor expression loss may partly reflect a reduction in stromal compartments [20]. These findings underscore the need to interpret miR profiling data in the context of tissue architecture.

Moreover, stromal overexpression of both miRs has been shown to promote neoangiogenesis and tumor growth, particularly in lung cancer, despite their tumor-suppressive role in epithelial tumor cells [28].

Collectively, these observations indicate that the cluster can exert both tumor-suppressive and tumor-supportive effects depending on tissue context, cell type, and microenvironmental conditions [20, 21, 28]. The cell-type-specific expression pattern also has diagnostic implications. In prostate cancer tissue, downregulation of the cluster compared with benign tissue has been consistently demonstrated [5, 6, 14], supporting its incorporation into multimodal diagnostic strategies.

### Functional role in cancer cells

The miR-143/145 cluster is among the most extensively studied miR-based tumor suppressor systems. In tumor cells, its downregulation derepresses oncogenic signaling pathways, such as the RAS-MAPK axis [23], whereas increased expression in stromal compartments can exert pro-angiogenic effects [20]. This cell type-specific divergence shapes its overall functional impact.

Consistent downregulation has been reported across multiple malignancies, including colorectal, breast, renal, and bladder carcinomas, and is linked to regulation of central oncogenic pathways [3, 22, 29, 30]. In colorectal tumors, both miRNAs regulate key growth pathways by targeting KRAS, ERK5, and the Insulin-Like Growth Factor 1 Receptor (IGF1R), thereby influencing proliferative and metabolic programs [3, 18, 23]. In breast cancer, cooperative effects between the ErbB2 Receptor Tyrosine Kinase 3 (ERBB3; also known as Human Epidermal Growth Factor Receptor 3: HER3) and c-MYC have been demonstrated, effectively limiting proliferation and invasion [13, 19]. In renal cell carcinoma, the cluster targets Hexokinase 2 (HK2) and modulates apoptosis and metabolic signaling [30].

The regulation of metabolic enzymes further links the miR-143/145 cluster to tumor-associated metabolic programs. In several tumor models, miR-143/145-dependent repression of metabolic regulators, including hexokinase 2 (HK2), has been reported, indicating that the cluster may influence central pathways of cellular energy metabolism [30]. Because HK2 is a key enzyme controlling glycolytic flux, its deregulation has been associated with tumor-related metabolic reprogramming. Although these observations were primarily described in other tumor entities, they suggest that loss of miR-143/145 may contribute not only to proliferative and invasive phenotypes but also to metabolic alterations relevant for tumor progression. The potential contribution of such mechanisms to prostate cancer biology remains an area requiring further dedicated investigation.

In bladder cancer, the miR-143/145 cluster controls the Plasminogen Activator Inhibitor 1 (PAI-1; also known as Serpin Family E Member 1: SERPINE1)-mediated invasion axis and exerts metastasis-suppressive effects [29]. In addition, the cluster interacts with angiogenic and stromal networks. While tumor-suppressive effects predominate in tumor cells, elevated expression in stromal compartments, particularly in lung cancer, can activate pro-angiogenic and tumor-promoting programs [28].

Downregulation of the cluster in malignant tissues is rarely attributable to mutations but instead predominantly results from epigenetic silencing, Ras Responsive Element Binding Protein 1 (RREB1)-dependent repression, or disturbances in p53 regulation and miRNA biogenesis [4, 15–17].

Restoration of miR-143/145 expression reduces tumor growth, metastasis, and chemoresistance in numerous preclinical models. Re-expression enhances sensitivity to 5-fluorouracil, gemcitabine, and targeted therapies in colorectal and pancreatic models [31, 32].

In triple-negative breast cancer, miR-145 modulates epithelial-mesenchymal transition (EMT)-associated factors and reduces invasiveness and stem cell-like features [33].

These findings have stimulated the development of therapeutic strategies, including chemically modified miR mimetics and nanoparticle- and polymer-based vector systems, which are currently in preclinical and translational development [34, 35].

From a diagnostic perspective, miR-143 and miR-145 are increasingly incorporated into multimarker-based signatures for tumor classification, assessment of progression dynamics, and modeling of therapeutic response. Studies in prostate, bladder, and colorectal cancer demonstrate that the cluster provides robust prognostic and diagnostic information [5–8].

**Fig. 1:**
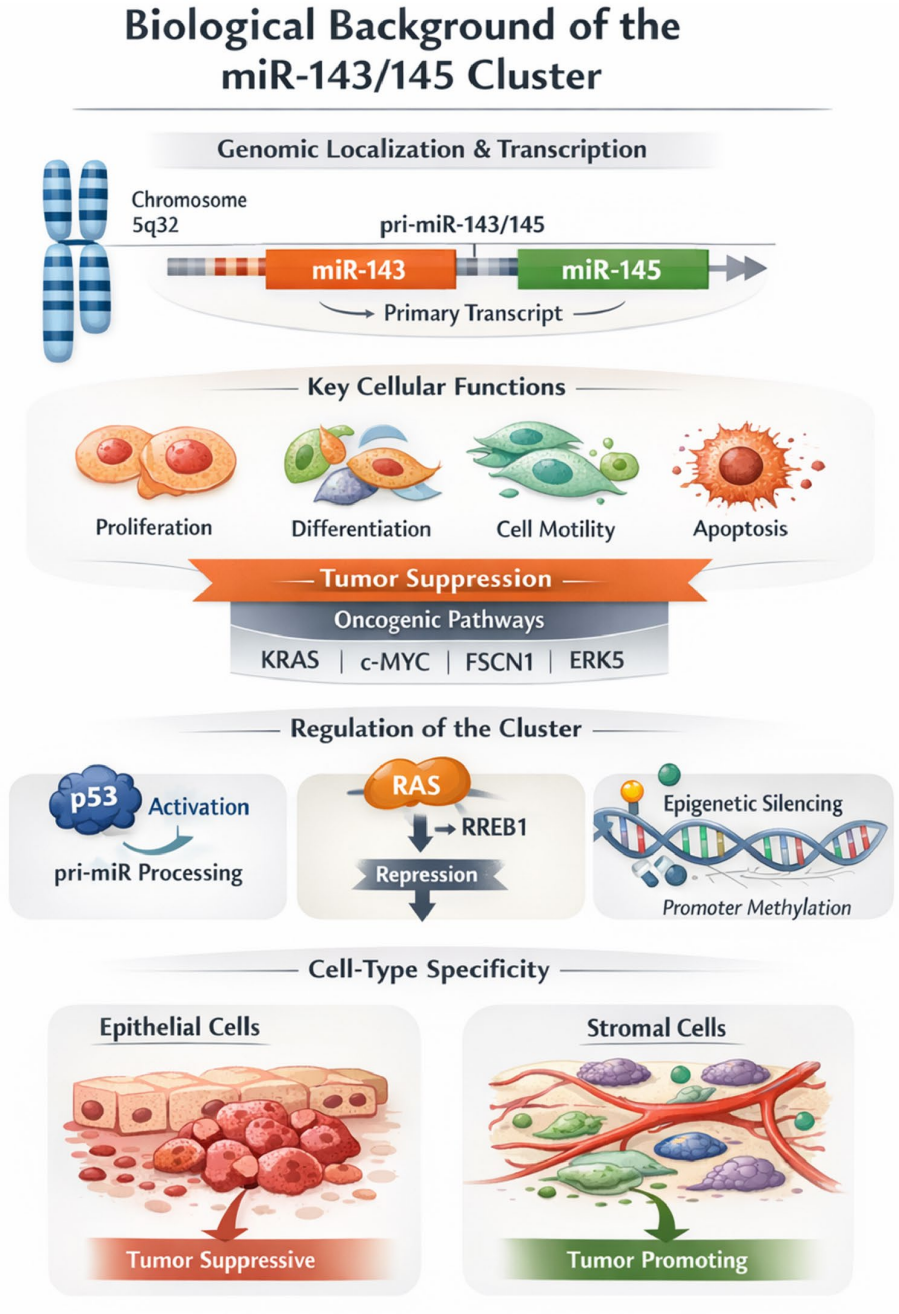
Biological background and regulatory network of the miR-143/145 cluster. Genomic organization, regulatory control, and functional roles of the miR-143/145 cluster. The cluster is located on chromosome 5q32 and transcribes a common primary transcript (pri-miR-143/145), which is processed into the mature miRs miR-143 and miR-145. Both miRs regulate key cellular processes, including proliferation, differentiation, cell motility, and apoptosis, thereby exerting tumor-suppressive effects by repressing oncogenic targets such as KRAS, ERK5, c-MYC, and FSCN1. Multiple regulatory mechanisms, including p53-dependent activation of pri-miR processing, repression through the RAS-MAPK pathway via RREB1, and epigenetic silencing through promoter methylation control cluster expression. In addition, the expression of miR-143/145 is strongly cell type-specific, with higher levels in stromal cells and lower expression in epithelial tumor cells, contributing to context-dependent tumor-suppressive or tumor-promoting effects within the tumor microenvironment

Overall, the miR-143/145 cluster emerges as a broadly conserved regulator of central oncogenic networks across multiple tumor types (Fig. 1). Owing to the pronounced context-dependent interactions between epithelial tumor cells and stromal compartments, it holds substantial translational potential, which requires targeted delivery strategies, precise functional analyses, and prospective clinical validation.

## Role of miR-143/145 in the development of prostate cancer

### Expression changes in early stages

Multiple miRNA profiling studies document a marked downregulation of miR-143 and miR-145 in prostate carcinoma tissue compared with benign prostate tissue. Consistent downregulation of both miRs in primary tumors has been reproduced across sequencing, microarray, and multiplex platforms [5–7, 14]. In addition, a progressive decline in cluster expression has been described along the course of tumor progression from high-grade intraepithelial neoplasia to metastatic disease [36].

Loss of the miR-143/145 cluster represents an early and frequent event in epithelial tumor development, as supported by data from additional tumor entities. Pronounced downregulation of the cluster has been documented in breast, colorectal, renal, and bladder carcinomas [3, 4, 13, 22]. The combined deregulation of central suppressor elements disrupts p53-dependent control mechanisms, activates proliferative programs, and impairs apoptotic signaling pathways [3, 4].

However, the predominantly stromal localization of both miRs complicates the interpretation of bulk tissue data. Laser capture microdissection studies indicate that apparent tumor expression loss may partially reflect a reduction in stromal cell components [20]. Analyses of the tumor microenvironment further demonstrate that stromal structural alterations substantially contribute to observed expression patterns and do not necessarily reflect epithelial-specific deregulation [21]. It therefore remains unresolved to what extent cluster downregulation primarily reflects alterations within tumor cells or rather changes in the stromal compartment.

### Molecular networks in tumor initiation

Functional and genetic studies indicate that reduced expression of the miR-143/145 cluster lowers the threshold for malignant transformation of prostate epithelial cells. A central mechanism involves disruption of p53-dependent regulation. In the tumor context, p53-mediated enhancement of pri-miR processing [16] and transcriptional induction of miR-145 [17] are attenuated, resulting in diminished repression of proliferative and survival signals.

Epigenetic mechanisms further reinforce this effect. Methylation of the miR-145 promoter, particularly in combination with p53 mutations, leads to a pronounced loss of miR-145 expression [15]. In parallel, activation of the RAS-MAPK signaling pathway via RREB1 mediates transcriptional repression of the entire cluster [23]. Under conditions of increased RAS signaling activity, this mechanism establishes a feed-forward loop in which loss of miR-143/145 derepresses multiple proliferation-promoting effectors [27].

**Fig. 2:**
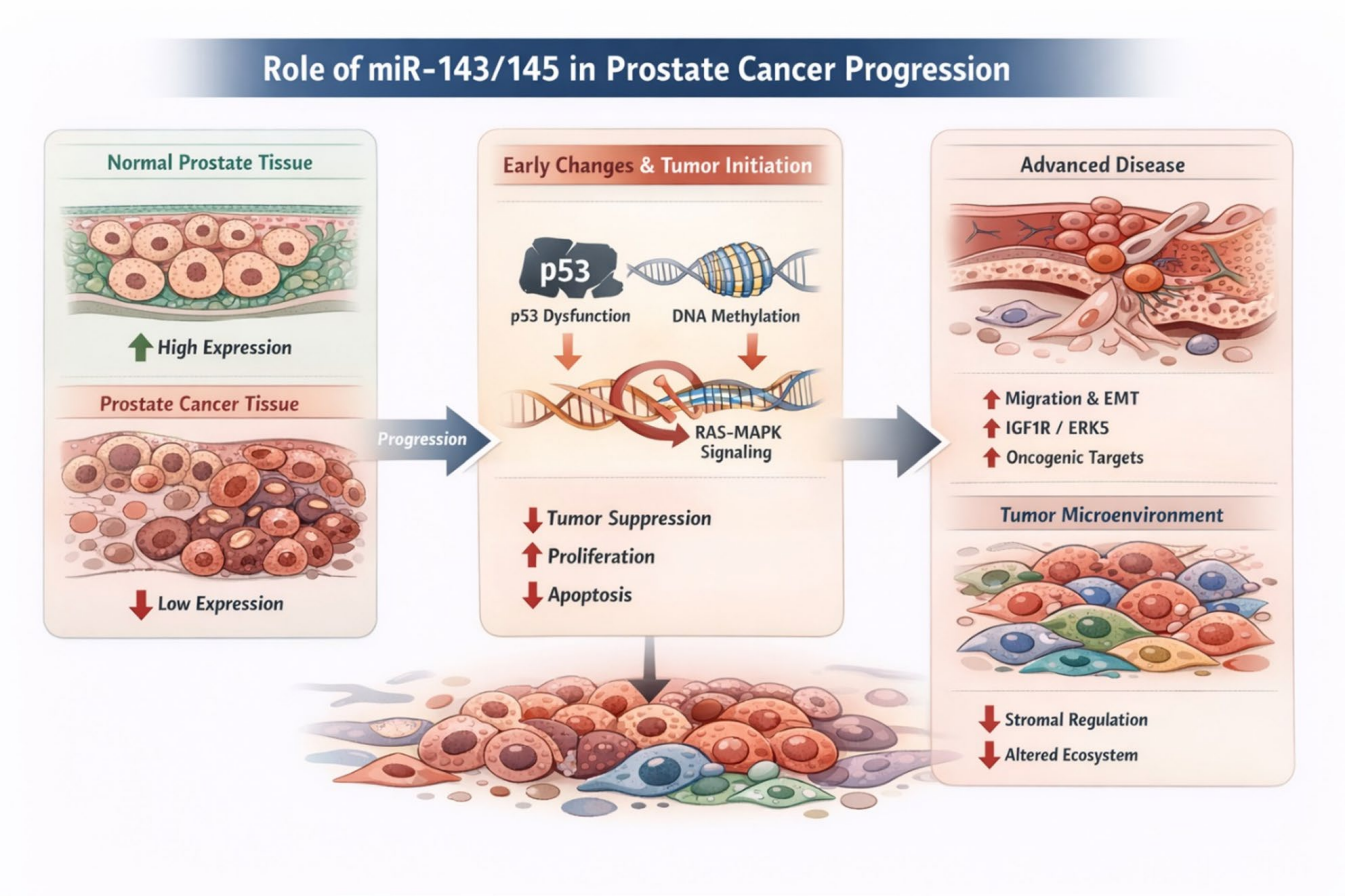
Role of the miR-143/145 cluster in early prostate cancer development. Schematic representation of expression changes and molecular mechanisms linking miR-143/145 downregulation to prostate tumor initiation and progression. In physiologic prostate tissue, miR-143 and miR-145 are expressed at higher levels, whereas a marked reduction of both miRs is consistently observed in prostate carcinoma. Early loss of the cluster is associated with disruption of tumor-suppressive regulatory networks, including attenuation of p53-dependent transcriptional control and epigenetic silencing through promoter methylation. Concurrent activation of RAS-MAPK signaling contributes to transcriptional repression of the cluster via RREB1, establishing a feed-forward regulatory loop that enhances proliferative signaling. Derepression of oncogenic targets such as IGF1R and ERK5 promotes proliferation, survival, and transformation-associated pathways, thereby lowering the threshold for malignant transformation. In addition, the predominantly stromal localization of miR-143/145 complicates interpretation of bulk expression data, as reduced stromal cell content in tumor tissue may contribute to the apparent downregulation observed in profiling studies

Under physiological conditions, the cluster directly regulates several oncogenic targets, including the IGF1 receptor [18], multiple RAS-dependent effectors [3], and the MAPK-related kinase ERK5 [24]. Loss of this regulatory control promotes proliferative, anti-apoptotic, and transformation-associated programs, creating a permissive molecular environment that facilitates tumor development (Fig. 2). However, cluster loss alone is not considered a sufficient driver of malignant transformation.

## Role in progression, invasion, and metastasis

### Control of proliferation and apoptosis in prostate cancer cells

Multiple functional studies in prostate cancer cell lines demonstrate a pronounced tumor-suppressive effect of miR-143 and miR-145. Experimental restoration of miR-143 results in marked inhibition of proliferation and increased cell death, mediated in part through direct repression of the MAPK-related kinase ERK5 [24]. In corresponding mouse models, re-expression of miR-143 leads to a significant reduction in tumor progression [24].

For miR-145, suppression of the pro-survival factor BNIP3 has been identified as a central mechanism underlying growth inhibition and enhancement of hypoxia-induced apoptosis. Restoration of miR-145 reduces proliferation, augments apoptotic responses under hypoxic conditions, and inhibits tumor growth in vivo [37].

The cluster collectively regulates additional factors relevant to tumor progression. The Golgi phosphoprotein GOLM1, whose overexpression is associated with increased migration and invasion in prostate cancer, is directly controlled by miR-143/145. Re-expression of the cluster results in a marked reduction of cell migration and invasion [38].

Taken together, these findings support the concept that both miRs exert anti-proliferative and pro-apoptotic functions in prostate epithelial cells. These effects are primarily mediated by the repression of central growth and survival regulators, including ERK5, BNIP3, and GOLM1 [4, 24, 37, 38].

### Regulation of EMT, cell motility, and bone metastasis

Control of EMT and cell motility represents a major mechanism by which the miR-143/145 cluster influences prostate cancer progression. A central component is the regulation of the actin-binding protein FSCN1, which is required for the formation of invasive filopodia and thereby promotes aggressive cellular behavior. Restoration of miR-145 results in a marked reduction of proliferation, migration, and invasion [26].

Another key mechanism involves the regulation of the adhesion- and signaling-adaptor HEF1/NEDD9. miR-145-dependent repression of this axis promotes re-epithelialization, characterized by increased E-cadherin and decreased N-cadherin and vimentin expression. In preclinical models, this regulation leads to significant inhibition of invasion, particularly bone invasion [25].

At a broader level, EMT-associated alterations and metastatic programs are modulated by miR-143 and miR-145. Both miRs are components of a molecular signature that correlates specifically with the presence of bone metastases in prostate cancer [39]. This indicates that the cluster regulates not only individual EMT effectors but is also linked to higher-order metastatic phenotypes.

Cell state-dependent effects further illustrate the functional complexity of the cluster. In prostate cancer stem cell populations, miR-143 expression is reduced, whereas it increases in differentiating tumor cells. Under certain differentiation conditions, miR-143 may even exert metastasis-promoting effects by repressing FNDC3B [40]. This example highlights the pronounced context dependency of miR-mediated regulation.

Overall, a consistent pattern emerges: miR-145 acts as a dominant inhibitor of EMT and invasion, primarily by regulating FSCN1, HEF1/NEDD9, and other motility-associated factors, such as GOLM1 [25, 26, 38]. In contrast, miR-143 displays a more complex, cell state-dependent functional profile that may include both anti-invasive and potentially metastasis-promoting effects [39, 40].

### KRAS axis and chemoresistance

Regulation of the KRAS axis constitutes another central mechanism by which miR-143 modulates prostate cancer progression. Direct repression of KRAS leads to a marked decrease in proliferative and migratory activity and increases sensitivity to docetaxel [41]. Suppression of KRAS underscores the close integration of miR-143 in the control of RAS-dependent growth signaling.

In addition, miR-143 and miR-145 regulate multiple components of the RAS-MAPK pathway, including ERK5 and additional downstream effectors relevant to proliferation [3, 18]. Repression of these signaling axes reduces proliferative capacity and attenuates pro-invasive programs, as observed in both cell culture systems and preclinical animal models [3, 24]. The cluster thus functions as a central integrative node of oncogenic RAS signaling.

Another important aspect concerns the modulation of therapy-associated resistance mechanisms. Interference with the RAS-MAPK pathway affects apoptotic signaling, metabolic adaptation, and stress response pathways, all of which are critical for survival under chemotherapeutic pressure [4, 18]. Restoration of cluster function can therefore reduce intrinsic cellular viability and enhance the efficacy of cytotoxic therapies.

In the context of docetaxel, preclinical data demonstrate that KRAS repression by miR-143 enhances cytotoxicity and induces a synergistic response [41]. Given that miR-143 additionally regulates further RAS-associated effectors, this mechanism may have implications for combinatorial therapeutic strategies aimed at overcoming or preventing chemoresistance [24].

**Fig. 3:**
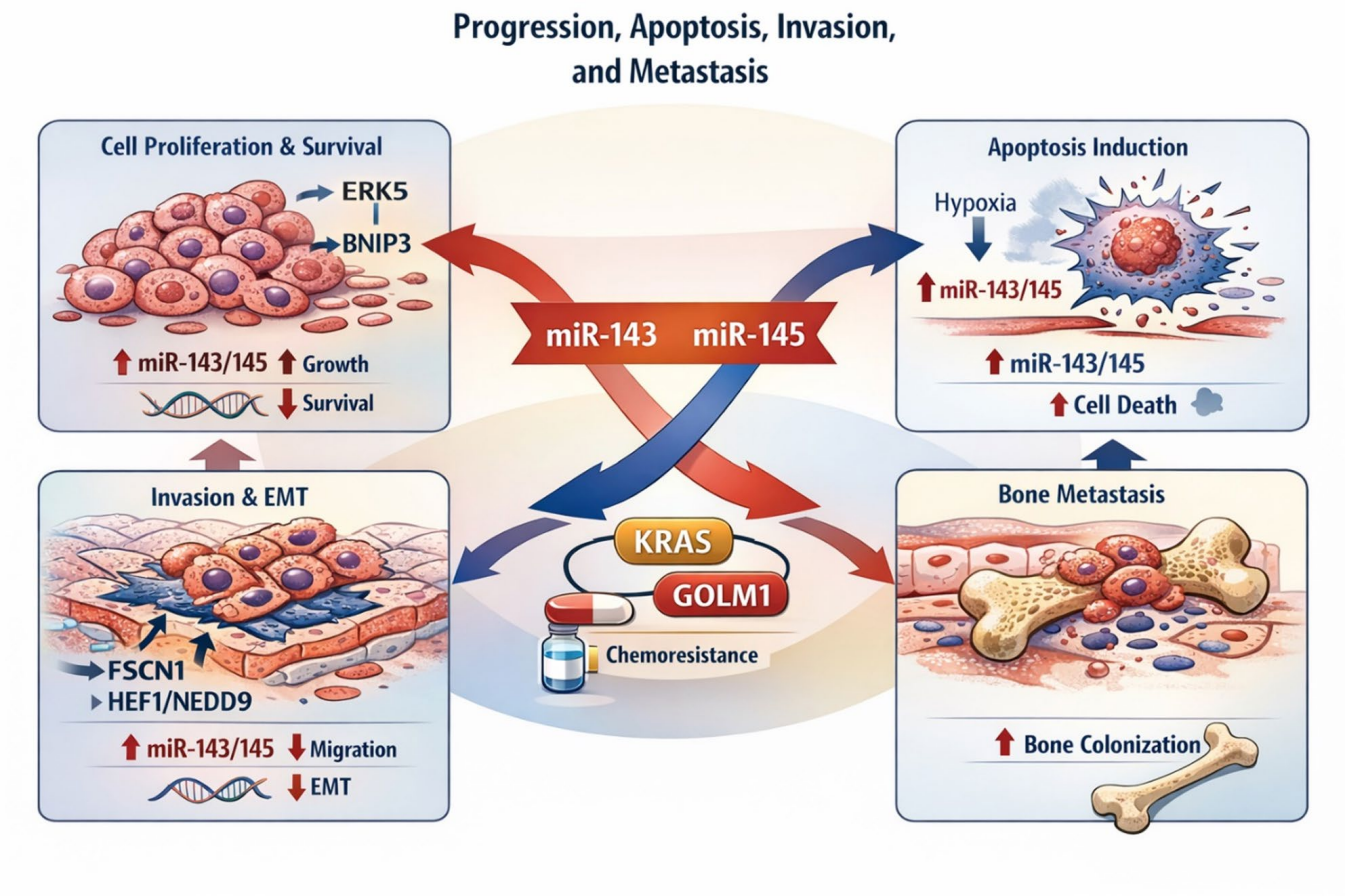
Role of the miR-143/145 cluster in prostate cancer progression, invasion, and therapy response. Schematic representation of the major mechanisms by which miR-143 and miR-145 regulate prostate cancer progression. Restoration of miR-143 suppresses proliferative signaling by repressing ERK5 and KRAS, whereas miR-145 promotes apoptosis under hypoxic conditions by targeting BNIP3. Both miRNAs coordinately inhibit migration and invasion by regulating cytoskeleton-associated and adhesion-related proteins, including FSCN1, HEF1/NEDD9, and GOLM1, thereby limiting EMT and metastatic dissemination. In addition, repression of KRAS-dependent signaling pathways enhances docetaxel sensitivity and reduces chemoresistance. Together, these mechanisms illustrate the central tumor-suppressive role of the miR-143/145 cluster in controlling proliferation, apoptosis, invasive behavior, and therapeutic response in prostate cancer cells

In summary, the miR-143/145 cluster functions as a central negative regulator of RAS-dependent signaling pathways and influences both tumor progression and response to systemic therapies (Fig. 3). Targeted reactivation of the cluster, therefore, represents a dual therapeutic approach, combining suppression of proliferative oncogenic programs with enhancement of conventional chemotherapeutic efficacy [3, 18].

## Potential cooperative effects of miR-143 and miR-145

Because miR-143 and miR-145 are transcribed as a cluster and frequently regulate related oncogenic programs, a cooperative functional effect appears biologically plausible. Experimental evidence from nonprostatic tumor models indicates that both miRNAs can act in a coordinated manner, either by converging on shared target transcripts such as ERBB3 or by repressing distinct components within the same signaling networks, particularly growth factor receptor, MAPK, and p53-associated pathways [19]. In prostate cancer, direct evidence for true cooperativity in the strict mechanistic sense remains limited; however, the reported cluster-dependent repression of GOLM1 and the recurrent convergence on motility, invasion, and growth-related pathways support the concept of a coordinated rather than purely independent tumor-suppressive action [38]. Thus, the available data support a cautious interpretation that miR-143 and miR-145 may exert cooperative biological effects in prostate cancer, although this requires more direct validation in cell-type-specific experimental models [3].

## Diagnostic relevance of miR-143/145

The diagnostic application of miRs has evolved from a conceptual framework to an extensively investigated field with clearly defined clinical perspectives. At the tissue level, characteristic miR signatures have been identified across numerous solid tumors, enabling discrimination of tumor entities and correlating with both prognosis and therapeutic response [11, 42]. Building on these findings, circulating miRs in serum and plasma have been characterized as stable, RNase-resistant biomarkers that reliably distinguish cancer patients from healthy individuals and therefore represent minimally invasive tools for cancer detection [43].

Reviews indicate that panel-based signatures comprising multiple circulating miRs achieve higher diagnostic accuracy than single markers and are suitable for early detection, staging, and longitudinal monitoring of various malignancies, including colorectal, pulmonary, breast, and urological cancers [44–46]. In addition, meta-analyses and clinical cohort studies suggest that perioperative changes in circulating miRs may provide information on resectability, minimal residual disease, and early recurrence, potentially complementing established markers such as Carcinoembryonic Antigen (CEA) or Prostate Specific Antigen (PSA) [47].

At the same time, methodological analyses demonstrate that pre-analytical variables, platform heterogeneity, normalization strategies, and lack of standardized cutoff definitions substantially limit comparability between studies and currently restrict broad clinical implementation [45, 46, 48]. Overall, available evidence supports the concept that miR-based diagnostics may provide clinical benefit primarily within multimodal strategies that integrate established serological and imaging modalities [11, 44, 45, 47].

The diagnostic potential of miR-143 and miR-145 has also been repeatedly evaluated in prostate cancer. Both miRNAs have been identified as components of panels that reliably distinguish prostate carcinoma from benign prostate tissue [5]. Downregulation of the cluster has been confirmed in independent cohorts using deep sequencing approaches [14] and reproduced across multiplatform profiling studies [6, 7]. Furthermore, signatures of small non-coding RNAs, including miR-143/145, have been shown to contain diagnostic and prognostic information and to contribute to discrimination between indolent and aggressive tumor courses [6].

A systematic review emphasizes that miR-143 and miR-145 have been investigated as components of tissue-based diagnostic panels in multiple studies, with performance strongly influenced by platform, normalization method, and panel composition. Available data suggest that the cluster is most informative within multimarker signatures, whereas its performance as a single marker appears limited [49].

For circulating variants in serum, plasma, or urine, data remain less robust and less consistent. Individual studies report differential expression of miR-145 in liquid biopsy settings [6], while methodological analyses addressing miR delivery and analytical platforms underscore existing technical challenges [34, 35]. Clinical implementation, therefore, requires larger prospective studies and standardized analytical infrastructures.

Interpretation of tissue-based diagnostic data must consider that miR-143/145 are predominantly expressed in stromal cell types. Analyses of tissue architecture indicate that an apparent reduction in cluster expression in tumor samples may partially reflect decreased stromal content [20]. Complementary studies demonstrate that structural alterations of the tumor microenvironment substantially contribute to observed expression patterns [21]. Accordingly, downregulation of the cluster frequently reflects not only changes within tumor cells but also profound shifts in tissue organization.

## Therapeutic approaches: miR replacement and targeting

### Preclinical substitution of miR-143/145 in prostate cancer

Several preclinical studies have investigated whether restoration of the miR-143/145 cluster in prostate cancer cells confers therapeutic potential. In mouse models, administration of miR-143 mimetics resulted in a marked reduction of tumor growth, partly mediated through inhibition of the ERK5 signaling pathway [24]. Similarly, miR-145 mimetics have been shown to exert anti-proliferative effects in prostate cancer cells, enhance hypoxia-induced apoptosis, and reduce tumor growth in vivo by repressing the pro-survival factor BNIP3 [37].

Additional investigations demonstrate that re-expression of the miR-143/145 cluster decreases expression of the Golgi phosphoprotein GOLM1, thereby reducing migration and invasion of prostate cancer cells [38]. Restoration of miR-145 further modulates key EMT-associated motility factors, including FSCN1 and HEF1/NEDD9, leading to pronounced inhibition of invasion, particularly bone invasion [25, 26]. Moreover, miR-143 substitution has been shown to suppress KRAS, limit proliferation and migration, and increase docetaxel sensitivity [41].

Chemically modified miR-143 oligonucleotides have been evaluated in additional preclinical systems as potential therapeutic tools and demonstrated antitumoral effects, supporting the feasibility of a miR-143-based replacement strategy [35].

### Vectors and drug delivery

Non-viral vector systems, including lipid nanoparticles, synthetic polymers, and ligand-conjugated constructs, currently constitute the principal platforms for therapeutic delivery of regulatory miRs [34, 50]. These approaches have been evaluated predominantly in preclinical models and early clinical trials for selected miRs, such as miR-34a. In contrast, no specific clinical development programs for miR-143 or miR-145 in the context of prostate cancer have been reported to date [34, 50].

Therapeutically oriented analyses indicate that miR-145, based on consistently reproduced tumor-suppressive effects in prostate, bladder, and renal carcinomas, represents a promising candidate for combinatorial strategies, including integration with androgen deprivation or targeted therapies [8]. This assessment is supported by preclinical data demonstrating robust repression of central oncogenic signaling pathways and motility-associated factors [37].

### Context-dependent risks and challenges

The stromal and vascular expression of the miR-143/145 cluster has significant implications for the safety of therapeutic replacement strategies. In specific tumor contexts, increased stromal expression can induce pro-angiogenic effects and promote tumor growth [28]. This finding highlights the risk of non-specific systemic administration, particularly in tissues such as vascular endothelium and smooth muscle cells, where the cluster is physiologically highly expressed [20, 21].

In addition, miR-143 has been shown to acquire metastasis-promoting properties in certain differentiation states of prostate cancer stem cell populations by regulating FNDC3B [40]. These observations underscore that miR-based interventions in heterogeneous tumors may yield context-dependent effects, potentially opposing one another.

Clinical translation, therefore, requires targeted, cell-type-specific delivery strategies, including ligand-conjugated vector systems or localized application approaches, to minimize off-target activity in stromal and vascular compartments. A detailed understanding of cell type-specific mechanisms of action, including potential pro-tumorigenic roles in defined subpopulations, remains essential [20, 21, 40]. Advances in non-viral vector platforms may substantially help address these challenges [34].

miR-based replacement strategies using miR mimetics to restore tumor-suppressive miRs such as miR-143 and miR-145 are associated with specific risks. A central limitation is off-target effects, in which introduced miRs regulate unintended transcripts, thereby inducing potentially undesirable biological consequences.

Off-target effects arise from partial sequence complementarity to non-intended miRs and may trigger alterations in metabolic, proliferative, or apoptotic programs in non-malignant tissues. This includes potential activation of pro-angiogenic signaling pathways in stromal cells [28].

Non-targeted overexpression in physiologically expressing tissues, such as vascular and smooth muscle compartments, may enhance angiogenic programs [28]. Preclinical data further indicate that elevated miR-143 levels can influence cell cycle-related networks [41]. Synthetic miR mimetics and their associated vector systems may elicit immunogenic reactions, as demonstrated in preclinical models [50].

The long-term systemic effects of miR mimetics remain insufficiently characterized. Although preclinical studies demonstrate antitumor efficacy, they do not allow a definitive assessment of potential long-term effects on the tumor microenvironment and non-malignant tissues.

## Prognostic significance

Multiple studies demonstrate pronounced prognostic relevance of miR-145 in prostate cancer. Low miR-145 levels in resection specimens have been associated with higher Gleason score, advanced tumor stage, larger tumor diameter, and shorter disease-free survival in clinical analyses [51]. In multivariate models, miR-145 was identified as an independent predictor of biochemical recurrence [51].

Comprehensive molecular profiling studies further support the prognostic significance of the cluster. miR-143 and miR-145 have been identified as components of multimodal miR signatures that predict more aggressive tumor courses [6], and similar associations have been confirmed in independent patient cohorts [7]. In addition, alterations in miR expression patterns, including miR-143/145, have been shown to correlate with progression from high-grade prostatic intraepithelial neoplasia to metastatic disease [36].

Meta-analyses and systematic evaluations of integrated datasets further indicate that reduced miR-145 expression is consistently associated with unfavorable histopathological parameters, accelerated tumor progression, and poorer clinical outcome [7, 8, 36]. This body of evidence underscores the robust prognostic value of miR-145 compared with miR-143, for which findings are less consistent. This discrepancy may reflect context-dependent mechanisms and functional particularities that become relevant in stem cell populations or early progression stages [40, 41].

Low miR-145 expression is consistently associated with adverse clinical course and reduced disease-free survival [51]. These findings support the incorporation of miR-145 into molecular risk models to stratify aggressive tumor phenotypes and support individualized therapeutic planning.

**Fig. 4:**
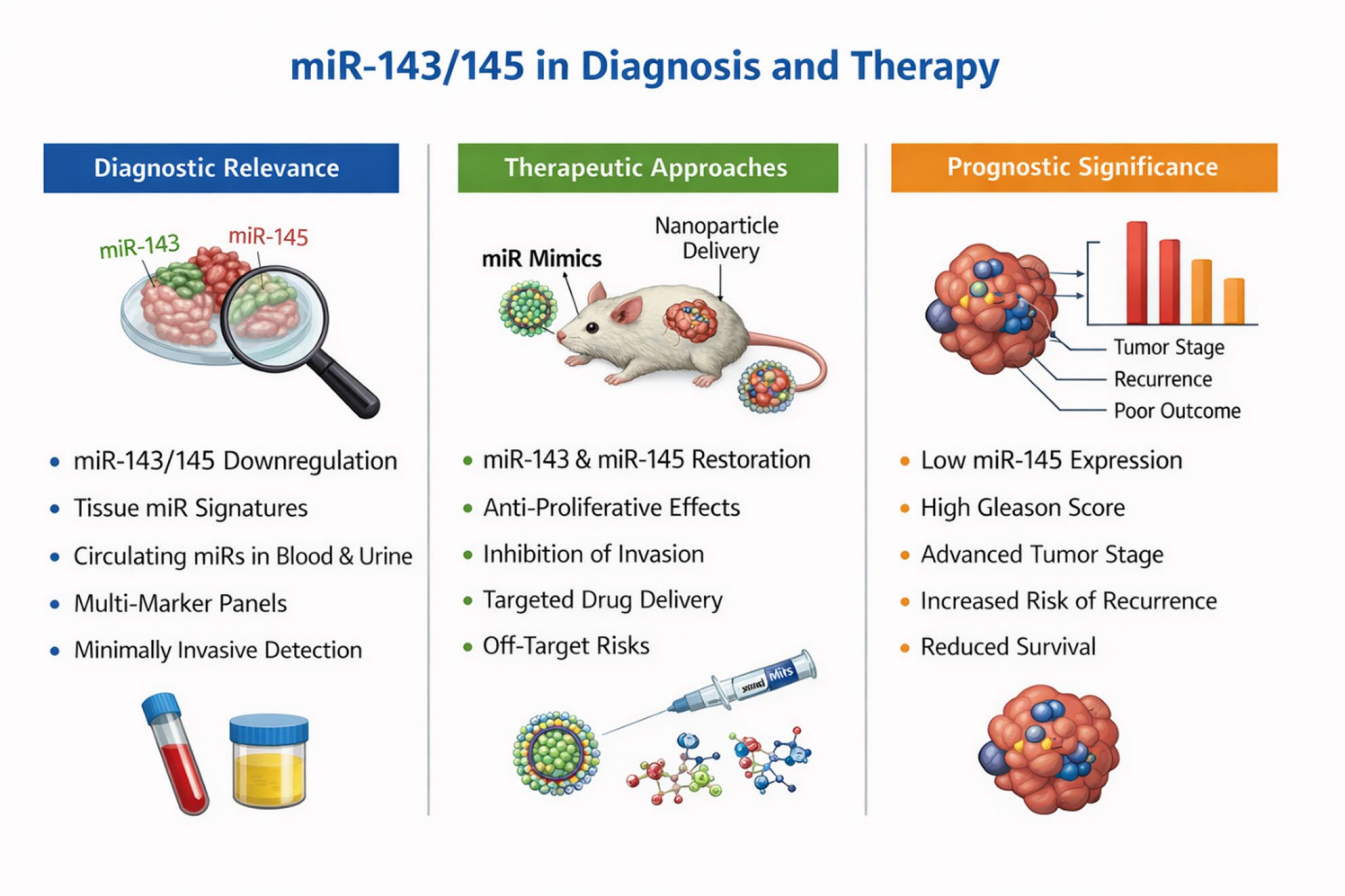
miR-143/145 in diagnosis and therapy. Overview of the diagnostic, therapeutic, and prognostic relevance of the miR-143/145 cluster in prostate cancer. The left panel illustrates diagnostic applications, highlighting the consistent downregulation of miR-143 and miR-145 in tumor tissue and their integration into tissue-based miR signatures, circulating miRs in blood and urine, and multimarker panels for minimally invasive cancer detection and disease monitoring. The central panel summarizes therapeutic strategies aimed at restoring tumor-suppressive miRs using miR mimics and delivery platforms, such as nanoparticle-based vectors, that inhibit proliferation and invasion but may also entail off-target risks. The right panel depicts the prognostic significance of the cluster, particularly the association of reduced miR-145 expression with adverse clinical parameters, including higher Gleason score, advanced tumor stage, increased risk of recurrence, and reduced survival. Together, these elements illustrate the translational potential of the miR-143/145 cluster for integrated diagnostic, therapeutic, and prognostic strategies in prostate cancer

Taken together, these findings illustrate the translational relevance of the miR-143/145 cluster across diagnostic, therapeutic, and prognostic contexts. The integrated clinical implications of these mechanisms are summarized in Fig. 4.

## Summary and outlook

Available experimental and translational data define a complex role of the miR-143/145 cluster in prostate cancer. Both miRs predominantly exert tumor-suppressive functions by regulating oncogenic signaling axes, including RAS-MAPK, ERK5, and KRAS, modulating p53-dependent growth inhibition with c-MYC repression, controlling EMT- and cytoskeleton-associated motility factors, and influencing apoptosis via BNIP3 [3, 4, 18, 24]. Loss of expression through p53 dysfunction, promoter methylation, or RAS-dependent repression facilitates malignant transformation and progression [15, 16, 23, 27].

Due to pronounced stromal and vascular expression, context-dependent pro-angiogenic effects may occur, particularly in lung cancer [20, 21, 28]. Such context specificity also applies to tumor cell subsets, including prostate cancer stem cells, in which miR-143 may promote metastasis [40].

From a diagnostic perspective, miR-143 and miR-145 contribute to multimarker RNA signatures that distinguish benign from malignant lesions and provide insights into tumor aggressiveness. Due to their predominant stromal localization, their value as single markers is limited, whereas combination with additional miRs or molecular parameters increases robustness [5–7, 14]. Prognostically, reduced miR-145 expression consistently correlates with adverse outcomes, early progression, and increased recurrence risk [6, 7, 36, 49, 51].

Therapeutically, preclinical models demonstrate that restoration of miR-143/145 suppresses proliferation, reduces invasiveness, and enhances docetaxel sensitivity. The therapeutic potential of restoring miR-143/145 has been investigated in several preclinical systems, including in vitro cell culture models and in vivo tumor models. In prostate cancer cell lines, re-expression of miR-143 or miR-145 by transfection with synthetic miR mimetics suppresses proliferation, migration, and invasion by regulating targets such as ERK5, FSCN1, HEF1/NEDD9, and GOLM1 [24, 26, 38]. In addition, xenograft mouse models have demonstrated that restoring miR-143 significantly reduces tumor growth and progression in vivo by inhibiting ERK5 signaling [24]. Further functional studies show that miR-143-mediated repression of KRAS enhances prostate cancer cells’ sensitivity to docetaxel [41]. Together, these preclinical models provide experimental evidence that reactivation of the miR-143/145 cluster can interfere with oncogenic signaling pathways and increase therapeutic vulnerability of prostate cancer cells.

While miR-145 shows consistent tumor-suppressive effects, miR-143 displays context-dependent and partially opposing functions in specific subpopulations [24, 25, 38, 41]. Clinical translation requires cell type-specific delivery strategies to minimize stromal off-target effects [20, 21, 28].

These findings suggest several clinical applications. Diagnostically, miR-143/145 may be integrated into multimodal panels for early detection and classification of aggressiveness. Prognostically, miR-145 appears suitable for incorporation into clinical risk scores, including prediction of biochemical recurrence after surgery. Therapeutically, miR-145 represents a candidate for replacement strategies, particularly in combination with androgen deprivation, chemotherapy, or targeted inhibitors. Implementation depends on reliable tumor cell-specific vector systems, as well as nanotechnological platforms, ligand-conjugated delivery systems, and locally applicable approaches.

Future investigations should prioritize cell-type-specific analyses of human prostate tissue, the optimization of tumor-targeted vector systems for miR-145-based therapies, and the prospective validation of diagnostic and prognostic signatures. On this basis, the biology of the miR-143/145 cluster may support more precise, biology-driven diagnosis and treatment of prostate cancer.

## Data Availability

No datasets were generated or analysed during the current study.
